# Preparation and Characterization of Vitamin D3-Based Binary Amorphous Systems

**DOI:** 10.3390/foods14081321

**Published:** 2025-04-11

**Authors:** Xiaoshuo Zhao, Xuemei Wang, Qiuyang Wu, Yiyang Cao, Xuening Song, Yingting Luo, Zisheng Luo, Jingwen Liu, Hao Zhang

**Affiliations:** 1 College of Food Science and Nutritional Engineering, China Agricultural University, Beijing 100083, China; 2021310060314@cau.edu.cn (X.Z.); sherry_xuemei@163.com (X.W.); 2021312100122@cau.edu.cn (Q.W.); yiyangcao02@outlook.com (Y.C.); 2020306100616@cau.edu.cn (X.S.); 2022306100208@cau.edu.cn (Y.L.); 2 College of Biosystem Engineering and Food Science, Zhejiang University, Hangzhou 310058, China; luozisheng@zju.edu.cn; 3 Department of Pharmacy, School of Medicine, University of Electronic Science and Technology of China, Chengdu 610072, China

**Keywords:** vitamin D3, L-arginine, amorphous, solubility

## Abstract

Vitamin D3 (VD3) is an essential nutrient for human health that plays a key role in bone health and immune regulation. However, VD3 deficiency has become a common issue worldwide due to insufficient daily intake and inadequate conversion from sunlight exposure. The relatively poor aqueous solubility of VD3 is one of the major challenges in the development of oral supplements and functional foods, since it usually results in low oral absorption. In this study, a total of 11 potential binary systems were prepared by solvent evaporation. The binary amorphous system of VD3 and L-arginine (ARG) has been found to be the most promising binary system, since the VD3–ARG system can significantly improve the solubility of VD3, with an 80-fold enhancement relative to neat crystalline VD3. The amorphization of the VD3–ARG binary system was confirmed and the morphology was observed. Molecular interactions between VD3 and ARG were mainly attributed to hydrogen bonding, and three specific bonding sites were revealed. Furthermore, superior dissolution behavior was observed in the VD3–ARG binary amorphous system compared to the neat VD3. A significantly higher saturation level was achieved and the saturation maintained for the desired period. Overall, this study developed a promising formulation strategy to enhance the solubility of VD3, which can be further applied in functional foods for VD3 supplements.

## 1. Introduction

Vitamin D is an essential nutrient with two active forms, i.e., vitamin D2 (ergocalciferol) and vitamin D3 (VD3, cholecalciferol). Among these two active forms, VD3 has been confirmed to be more effective in animal nutrition compared to vitamin D2 [1]. Specifically, VD3 plays a crucial role in promoting bone mineralization [2], regulating the immune system [3], reducing the incidence of cancers [4,5], and improving mental health [6,7].

VD3 is primarily derived from two sources, i.e., dietary intake and endogenous synthesis. To achieve the health benefits of vitamin D, the serum concentrations of 25-hydroxyvitamin D, which is the main active metabolite of VD3, are required to reach a level above 75 nmol/L (or 30 ng/mL) [6]. The recommended daily intake of VD3 varies across populations: infants (0–12 months) require 400 IU (10 μg), children and adults (1–70 years) 600 IU (15 μg), and older adults (>70 years) 800 IU (20 μg) [8]. However, the daily intake of VD3 often falls short of the physiological requirements in the human body, and the endogenous synthesis of VD3 exclusively occurs when 7-dehydrocholesterol in the skin is converted to VD3 upon exposure to sunlight [9]. However, environmental factors and modern lifestyle changes frequently impede the adequate synthesis of VD3. Many individuals are unable to achieve the required levels of VD3 through dietary intake and endogenous synthesis, leading to a widespread global deficiency of VD3 [2]. Therefore, vitamin D-fortified functional foods and oral supplements have become essential for maintaining adequate levels of this nutrient and reducing associated health risks.

Commercially available vitamin D supplements are predominantly used in the pharmaceutical sector for patients who have already exhibited clinical symptoms [9]. However, the supplementation and fortification of VD3 in food formulation are limited due to the lipophilic nature of VD3, which leads to poor solubility in gastrointestinal fluids [9,10]. In order to overcome the solubility challenge of VD3, several techniques have been investigated, including nanostructured systems [11,12], embedding methods [13], and encapsulation techniques [14]. However, the application of these techniques in food products remains limited due to the relatively low loading capacity of VD3 and the complexity of scaling up production. Recent studies have highlighted the limitations of current VD3 delivery systems in terms of loading capacity. Nanostructured systems (e.g., liposomes and polymeric nanoparticles) typically achieve VD3 loading rates of 5–15% (*w*/*w*) [11,12,15]. The embedding method shows similar constraints, with most formulations demonstrating < 10% (*w*/*w*) loading efficiency due to molecular size mismatch and weak host–guest interactions [13]. Encapsulation techniques typically result in VD3 loading of less than 8% (*w*/*w*) in food-grade applications when considering process scalability and ingredient compatibility [14]. In contrast, amorphous systems could offer a unique advantage of high loading capacity (i.e., >50% *w*/*w* in binary formulations), as demonstrated in recent studies [16].

Amorphization is one of the well-known techniques to overcome the poor aqueous solubility issue [16]. Theoretically, the crystalline state is thermodynamically in a relatively stable low-energy state, while the amorphous form, which is characterized by a lack in long-range molecular order, exhibits higher internal energy and reactivity [17]. Therefore, active components in the amorphous form often show improved solubility and an increased dissolution rate compared to their crystalline counterparts. However, the main drawback of using neat amorphous components is their poor physical stability during processing and storage, which usually results in potential recrystallization and thus the loss of the solubility advantage [18]. In recent years, binary amorphous systems have been developed as a promising strategy to overcome this issue [19]. The stabilization mechanism of binary amorphous systems varies based on the co-former selection, including molecular interactions, intimate mixing, good miscibility and elevated glass transition temperatures [19]. Amorphization techniques have primarily been utilized in the development of pharmaceutical products in past decades. Recently, this formulation strategy has garnered increasing interest in the field of nutrient development. Wang et al. developed a curcumin–piperine binary amorphous system and found that the maximum saturated solubility of curcumin in the binary amorphous system was 4.27-fold and 3.21-fold higher than that of neat curcumin in 0.2 M phosphate buffer (pH = 6.8) and 0.1 M hydrochloric acid buffer (pH = 1.2), respectively [20]. Li et al. found that the apigenin–oxymatrine binary amorphous system showed significant advantages of achieving supersaturation and increasing dissolution rates compared to pure apigenin [21]. We have also applied the amorphization technique in enhancing the solubility of lutein and phytosterols [22,23], and the newly developed binary amorphous systems could significantly improve the dissolution behavior compared to the neat active components. To the best of our knowledge, no studies have been reported on binary amorphous systems of VD3. Therefore, this study aims to design and prepare VD3-based binary amorphous systems to overcome the solubility challenge.

The selection of co-former is crucial for the preparation of a desired binary amorphous system. To involve VD3-based binary amorphous systems into functional foods in the future, other functional nutrients may be considered as ideal co-formers. Sugar alcohols (e.g., mannitol, erythritol, sorbitol, lactitol) are low-calorie sweeteners that provide alternatives to traditional sugars. They aid in glycemic control and reduce caloric intake, which can be beneficial for managing obesity and diabetes [24]. Amino acids, including L-arginine (ARG), methionine, and phenylalanine, play crucial roles in protein synthesis, tissue repair, immune function, and hormonal regulation [25]. In addition, certain sugars and oligosaccharides, such as D-xylose and oligolactose, can function as prebiotics to promote a healthy gut microbiome [26]. Vitamins, including vitamin B3, play crucial roles in energy metabolism, DNA repair, and cell signaling, thereby contributing to overall cellular health [27]. Considering the potential benefits of the above-mentioned nutrients, a total of 11 co-former candidates were selected and screened for the preparation of VD3-based binary amorphous systems in this study.

The binary systems of VD3 and functional co-former candidates were prepared by solvent evaporation, and the potential improvement in the solubility was evaluated. The prepared binary amorphous systems were characterized by powder X-ray diffraction (PXRD), differential scanning calorimetry (DSC) and scanning electron microscopy (SEM). Fourier transform infrared (FT-IR) spectroscopy and molecular dynamics (MD) simulations were applied to gain insights into the molecular interactions. Dissolution tests were conducted to investigate the solubility and dissolution performance of VD3-based binary amorphous systems. Overall, this study aims to provide a novel binary amorphous formulation to improve the solubility of VD3.

## 2. Materials and Methods

### 2.1. Materials

VD3 was purchased from Heowns Biochem Technologies LLC (Tianjin, China). All co-formers used in the co-former screening section were purchased from Shandong Pingju Biotechnology Co., Ltd. (Jining, China). ARG used in the preparation of binary amorphous systems was obtained from Fuzhou Feijin Biotechnology Co., Ltd. (Fuzhou, China). Chromatography-grade methanol was purchased from Beijing Mreda Technology Co., Ltd. (Beijing, China), and analytical-grade anhydrous ethanol was purchased from Tianjin Zhiyuan Chemical Reagent Co., Ltd. (Tianjin, China).

### 2.2. Preparation of VD3–Co-Former Binary Systems

Potential VD3–co-former binary amorphous systems were prepared by solvent evaporation. The VD3 and the co-former were initially dissolved in ethanal and water, respectively. The mixed ethanal–water (83.3% *v*/*v*) solution was subsequently prepared at a VD3-to-co-former molar ratio of 2:1. Afterwards, the mixed solution was rotary evaporated at 60 °C with a RE-2000A solvent evaporator (Yarong Biochemical Instrument, Shanghai, China) until a powder was obtained. The samples were collected on a glass plate and further dried in an oven at 65 °C for 20 min to remove residual solvent. For comparison, the corresponding physical mixtures (PM) of VD3 and co-former were prepared by mixing two crystalline components at the same molar ratio.

### 2.3. Co-Former Screening by Water Solubility Measurements

Co-former screening was carried out by evaluating the water solubility of VD3 in binary systems. Water solubility was measured based on the following procedures: 15 mg VD3–co-former binary mixtures were added to 15 mL water (pH 7) to reach the saturation concentration of VD3. The powder samples were dissolved in a mechanical shaker at a speed of 200 rpm for 24 h at room temperature. Afterwards, the samples were filtered through a 0.22 μm aqueous membrane to remove the undissolved components, and the dissolved VD3 in the filtered solution was measured by high-performance liquid chromatography (HPLC).

The concentration of VD3 was analyzed using an LC-20A HPLC system (Shimadzu, Tokyo, Japan) equipped with a C18 column (4.6 × 250 mm, 5.0 μm). The mobile phase consisted of methanol and water (97.5:2.5, *v*/*v*) at a flow rate of 1 mL/min [28]. The wavelength was set at 264 nm, and the column temperature was set at 35 °C. To construct a standard curve for VD3, VD3 was accurately weighed and dissolved in anhydrous ethanol to prepare a master batch at a concentration of 100 μg/mL. A series of standard solutions of 0.05, 0.10, 0.50, 1.00, 5.00, 10.00, and 50.00 μg/mL were prepared by gradient dilution with anhydrous ethanol and water. The solvent composition of each standard solution was kept the same, and the volume ratio of anhydrous ethanol to water was 1:1. Using the above chromatographic conditions, a standard curve was constructed with the concentration as the horizontal coordinate and the peak area as the vertical coordinate. The linear regression equation of the standard curve was y = 285,216x + 3334.1 (R^2^ = 0.9995). Based on the corresponding standard curve and the peak area of the sample to be measured, the concentration of VD3 in water could be detected.

### 2.4. PXRD Measurements

PXRD patterns of crystalline VD3, crystalline ARG, VD3–ARG PM, and VD3–ARG binary amorphous system were measured using an X-ray diffractometer (Malvern Panalytical Inc., Westborough, MA, USA) equipped with a Cu-Kα ray radiation source. The tube voltage and amperage were set at 36 kV and 20 mA, respectively. Each pattern was collected over a 2θ range from 5° to 45°. The scanning speed was 10°/min, with a step size of 0.02°.

### 2.5. DSC Measurements

DSC analysis of crystalline VD3, crystalline ARG, VD3–ARG PM, and VD3–ARG binary amorphous system was obtained using a DSC-80 (Baxit, Potsdam, Germany). The reference substance chosen for the analysis was aluminum, and the nitrogen flow rate was 30 mL/min. The sample (approximately 5 mg) was placed in a confined aluminum crucible and heated from 30 °C to 260 °C at a heating rate of 10 °C/min [29].

### 2.6. SEM Measurements

Surface morphology of crystalline VD3, crystalline ARG, VD3–ARG PM, and VD3–ARG binary amorphous system were observed through an SU-3500 SEM (Hitachi High-Tech Corporation, Tokyo, Japan). The powder samples were sputter-coated with a gold layer and subsequently placed in the instrument [30]. Images were obtained at an accelerating voltage of 3.0 kV after adjusting to the appropriate focal length.

### 2.7. FT-IR Analysis

FT-IR spectra of crystalline VD3, crystalline ARG, VD3–ARG PM, and VD3–ARG binary amorphous system were obtained using a Spectrum 3 FT-IR spectrophotometer (PerkinElmer Inc., Waltham, MA, USA). KBr was used as the blank sample. The samples were mixed with KBr at a weight ratio of 1:100 and pressed into tablets for measurements. The tablet samples were scanned in the range of 4000 cm^−1^ to 400 cm^−1^ with an average of 16 scans and a resolution of 1 cm^−1^.

### 2.8. MD Simulations

The molecular models of VD3 and ARG were obtained from the PubChem database (https://pubchem.ncbi.nlm.nih.gov/, accessed on 27 January 2024). More than 5000 geometry optimizations were conducted using Forcite code (Materials Studio 2020), and the binary amorphous system of VD3 and ARG was constructed under a 30/60 number ration using the periodic boundary condition (PBC) cubic unit code. All simulations were performed with a COMPASS II force field [21]. The canonical ensemble (NVT) was used, and each simulation was performed for a period of 1 ns with a time step of 1.0 fs. Radial distribution function (RDF) analysis was carried out using equilibrium configurations.

### 2.9. Dissolution Tests

Powder dissolution tests were conducted by a magnetic stirrer (Jintan Science Analysis Instrument Co., Ltd., Changzhou, China). Powder samples containing a total of 30 mg VD3 were added to 30 mL of ethanol–water (20:80, *v*/*v*) solution at a medium temperature of 37 °C. The stirring speed was set at 300 rpm. At predetermined time points (1, 3, 5, 10, 20, 40, 60, 90, 120 min), samples of 0.3 mL were withdrawn and immediately replaced with the same volume of pre-warmed dissolution medium [31]. The collected samples were quantified by HPLC after filtration (0.45 μm).

### 2.10. Data Analysis

Data analysis and graphical representation were performed using OriginPro 2022 software (OriginLab, Northampton, MA, USA) and SPSS 26.0 (IBM Co., Armonk, NY, USA). The experiments were performed in triplicate. The results are reported as mean ± standard deviation (n = 3). A *p*-value below 0.05 (*p* < 0.05) was considered statistically significant.

## 3. Results and Discussion

### 3.1. Co-Former Screening

This study aims to improve the dissolution behavior of VD3 by developing binary amorphous systems. Several potential co-formers were screened, and the most effective co-former was selected by detecting the water solubility of the binary VD3–co-former systems. Since all the potential co-formers had high water solubility, the water solubility tests of binary systems mainly focused on the solubility improvement of VD3.

**Figure 1:**
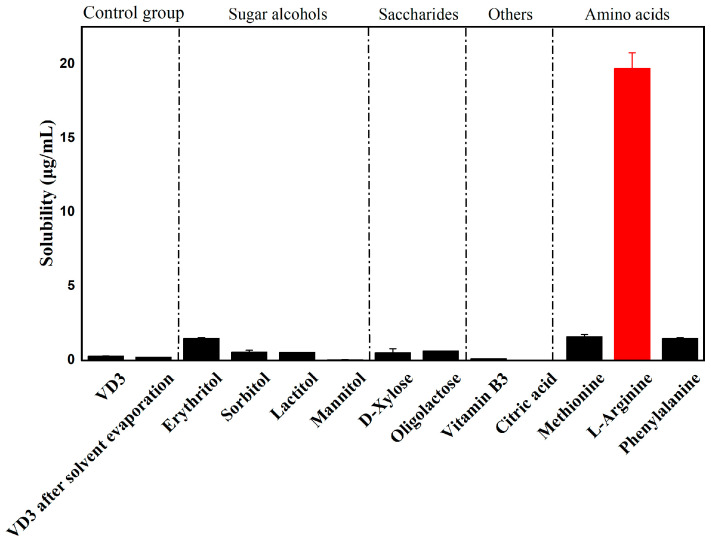
Water solubility of VD3 in potential binary systems with various co-formers.

A total of 11 co-formers were screened. These co-formers were categorized into sugar alcohols (mannitol, erythritol, sorbitol, lactitol), sugars (D-xylose and oligolactose), amino acids (phenylalanine, methionine, and ARG), and other co-formers (citric acid and vitamin B3). To dismiss the influence of the preparation process, the solubility of neat VD3 after solvent evaporation was detected. The water solubility of VD3 is presented in Figure 1. Neat VD3 exhibited a quite low water solubility (0.245 ± 0.007 μg/mL), and the solvent evaporation process did not improve its solubility. Among all the potential binary systems, the water solubility of VD3 increased significantly in the VD3–ARG system (19.697 ± 1.047 μg/mL), which was 80-fold higher than that of neat VD3. The VD3-ARG binary system was expanded to evaluate solubility under simulated physiological pH (3, 7 and 10), with the VD3-ARG system demonstrating enhanced solubility across conditions (Figure S1). Therefore, ARG could be regarded as an ideal co-former to prepare a binary amorphous system with VD3. The significant enhancement in solubility is usually attributed to the molecular interactions between two components [19]. ARG is a basic amino acid characterized by its strong alkalinity. The –NH groups of its guanidinium and amine moieties can act as hydrogen bond donors, and the carbonyl oxygen of its carboxyl group can serve as a hydrogen bond acceptor [32]. These functional groups of ARG facilitate the potential formation of hydrogen bonds or electrostatic interactions with specific sites in the VD3 molecule. Subsequently, the potential molecular interactions between ARG and VD3 could contribute to the enhancement in solubility.

### 3.2. Characterization of VD3–ARG Binary System

#### 3.2.1. PXRD of VD3–ARG Binary System

**Figure 2:**
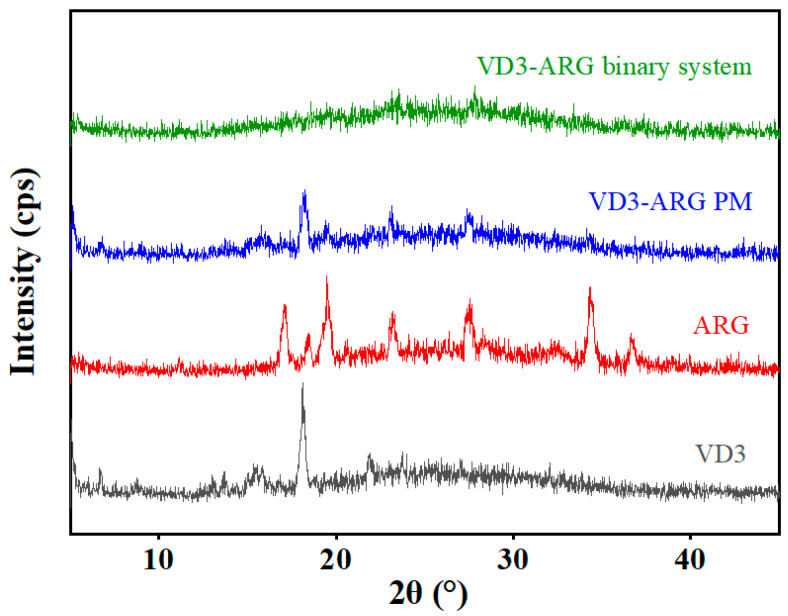
PXRD diffractograms of crystalline VD3, crystalline ARG, VD3–ARG PM, and the VD3–ARG binary system.

PXRD is widely used to characterize amorphous forms due to its efficiency, accuracy, and minimal material consumption [33]. The appearance of an amorphous halo pattern in the diffraction graph is indicative of a decrease in crystallinity. The diffractogram of neat VD3, ARG, the corresponding PM, and the solvent evaporated binary system are shown in Figure 2. Neat crystalline VD3 displayed strong characteristic diffraction peaks at 2θ angles of 15.6° and 18.0°, while ARG exhibited distinct diffraction peaks at 2θ angles of 16.6°, 18.3°, 19.6°, 23.3°, 27.8°, 34.1°, and 36.8°. The diffractograms of raw materials are consistent with previous literature [34]. In VD3–ARG PM, some of the characteristic diffraction peaks of crystalline VD3 and crystalline ARG were retained, albeit with relatively weaker intensity. In contrast, no obvious sharp diffraction peaks were observed in solvent evaporated in the VD3–ARG binary system, and a typical amorphous halo appeared. Therefore, the diffractograms confirmed that the amorphization of the VD3–ARG binary system was obtained after solvent evaporation. In terms of stability, the VD3-ARG binary amorphous system maintained physical stability during 6-month storage at 25 °C with PXRD patterns (Figure S2) confirming no recrystallization.

#### 3.2.2. DSC of VD3–ARG Binary System

**Figure 3:**
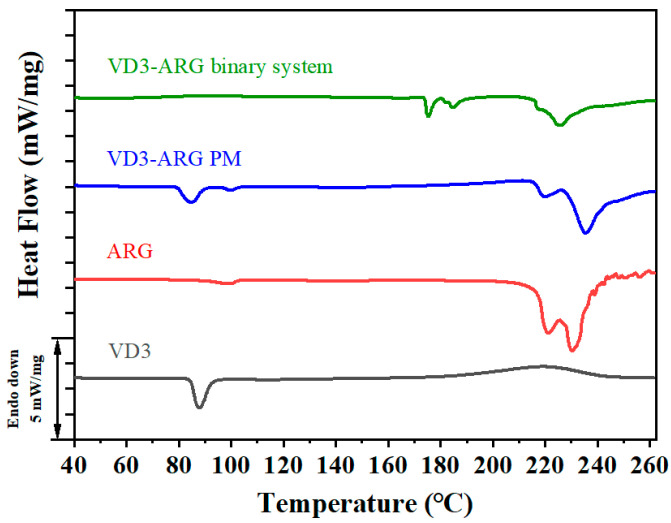
DSC thermograms of crystalline VD3, crystalline ARG, VD3–ARG PM, and the VD3–ARG binary system.

DSC is an efficient method for determining thermodynamic properties and for providing important information regarding amorphization. Sharp endothermic peaks in DSC curves typically indicate the presence of crystalline structures, while the absence of such peaks suggests an amorphous or highly disordered form [35]. According to the thermograms in Figure 3, the thermogram of crystalline VD3 presented an exothermic peak at 87.9 °C, indicating the melting point of crystalline VD3. The thermogram of crystalline ARG showed a low-intensity exothermic peak at 98.6 °C due to the loss of water, and the thermal decomposition of crystalline ARG was observed at around 210 °C. The thermogram of VD3–ARG PM retained the exothermic peaks that appeared in both crystalline VD3 and crystalline ARG thermograms. In contrast, the melting point signal of crystalline VD3 disappeared in the thermogram of the VD3–ARG binary system, which further confirmed the amorphization of VD3. In addition, some peaks appeared at around 170 °C, which may indicate the aggregation between two components, and the thermal decomposition above 200 °C was consistent with the decomposition behavior shown in the thermogram of crystalline ARG.

#### 3.2.3. Morphology of VD3–ARG Binary Amorphous System

**Figure 4:**
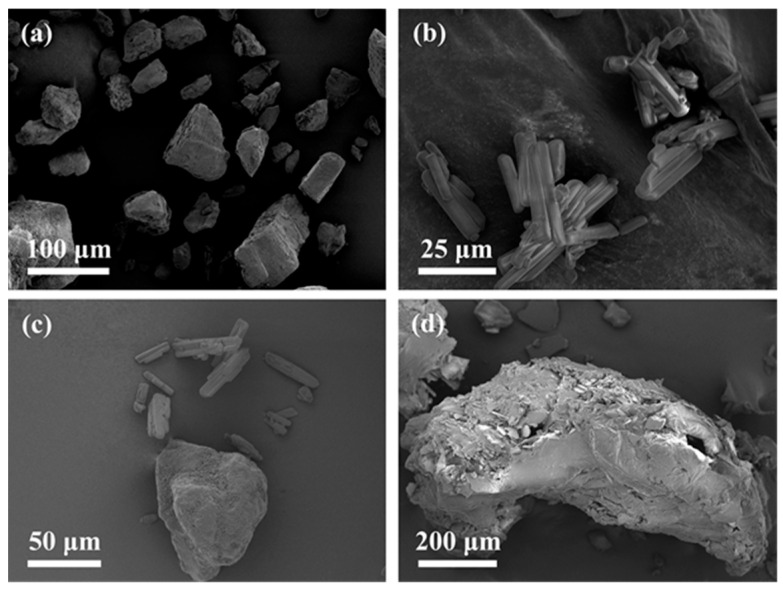
Morphology of crystalline ARG (**a**), crystalline VD3 (**b**), VD3–ARG PM (**c**), and the VD3–ARG binary system (**d**).

To detect the morphology changes in the preparation process of the binary system, the SEM images of crystalline VD3, crystalline ARG, VD3–ARG PM, and the VD3–ARG binary amorphous system are shown in Figure 4. The images indicate that crystalline ARG exhibited a large granular morphology (Figure 4a), while crystalline VD3 appeared as small bar-shaped crystals (Figure 4b). VD3–ARG PM retained the characteristic morphologies of both ARG and VD3 (Figure 4c), whereas the VD3–ARG binary system exhibited a different morphology. The VD3–ARG binary system formed larger and more irregular agglomerates compared to the neat crystalline VD3 and ARG. In detail, VD3–ARG agglomerates contained numerous internal spaces, which could potentially have increase the inner surface area and thereby enhance the solubility (Figure 4d).

### 3.3. Molecular Interactions of VD3–ARG Binary Amorphous System

#### 3.3.1. FT-IR of VD3–ARG Binary Amorphous System

**Figure 5:**
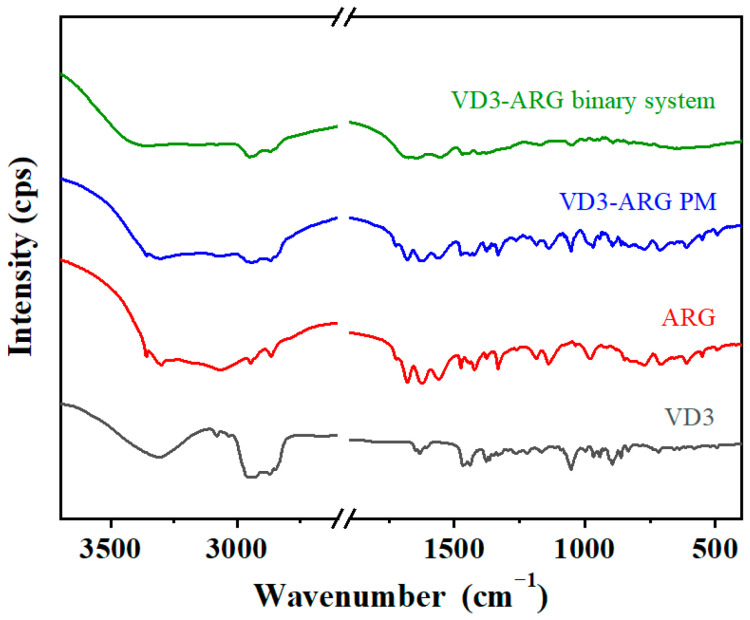
FT-IR spectra of crystalline VD3, crystalline ARG, VD3–ARG PM, and the VD3–ARG binary system.

FT-IR is widely used as a “fingerprint of molecules” [36], and therefore the changes in the FT-IR spectra can be applied to investigate possible interactions between two components. As shown in Figure 5, the spectrum of crystalline VD3 showed a relatively broad peak at 3338 cm^−1^ and a sharp peak at 1053 cm^−1^, which is attributed to the –OH and –C=O groups, respectively. The spectrum of crystalline ARG showed broad peaks of –NH and –OH groups in the range of 2900 cm^−1^ to 3300 cm^−1^, and the –C=O group exhibited a sharp peak at 1346 cm^−1^. The spectrum of VD3–ARG PM retained all these characteristic absorption peaks of crystalline VD3 and ARG, which implies that there were no significant interactions between the two substances in the PM. However, in the VD3–ARG binary amorphous system, the absorption peaks of –OH (3342 cm^−1^) and –C=O (1049 cm^−1^) from crystalline VD3 disappeared or weakened. Similar phenomena were observed in the typical ARG peaks of the –NH, –OH, and –C=O groups. The changes in these functional groups imply that the potential molecular interactions between VD3 and ARG occurred. Based on the structure of these functional groups, hydrogen bonding was anticipated to be the most likely interactions.

#### 3.3.2. MD Simulations of VD3–ARG Binary Amorphous System

MD simulations can complement experimental results by computationally modeling the motions and interactions within complex molecular systems, thereby providing deeper insights into intermolecular interactions. To further investigate the interacting sites between VD3 and ARG, RDF analysis was carried out in the VD3–ARG binary system.

**Figure 6:**
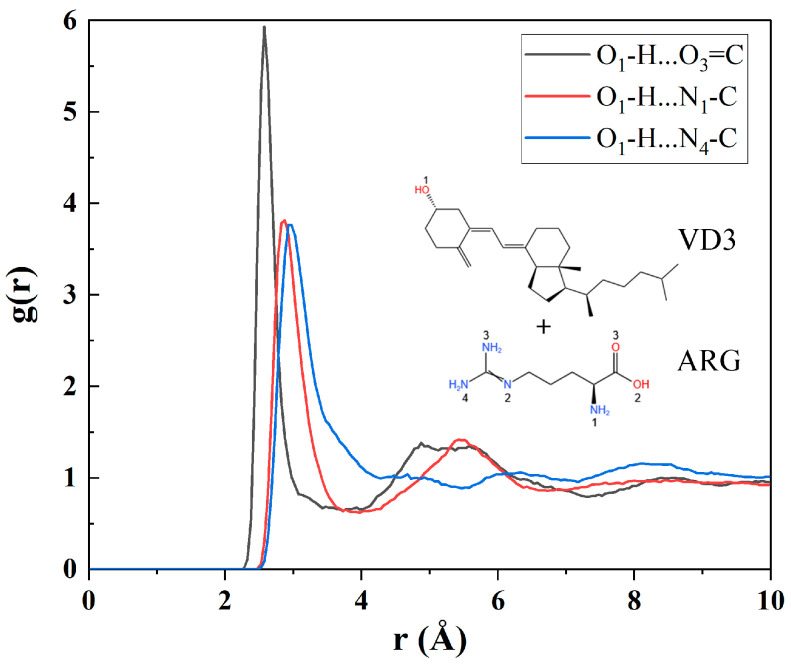
RDF analysis for hydrogen atoms of the VD3–ARG binary system.

Since hydrogen bonding was expected to be the main interaction between VD3 and ARG based on FT-IR spectra, the RDF curves of the VD3–ARG binary system focused on the hydrogen bonding area. In general, the g(r) peak of hydrogen bonding occurs within 3.5 Å [37]. In the RDF curves of the VD3–ARG binary system (Figure 6), there were three g(r) peaks located at 2.575 Å, 2.875 Å, and 2.925 Å. These peaks suggest that VD3 offered an O1-H group and formed three strong hydrogen bonds with ARG, i.e., O1-H…O3=C, O1-H…N1-C, and O1-H…N4-C. The results of the MD simulations are consistent with the FT-IR results and further clarify the hydrogen bonding sites between two components. Therefore, the strong hydrogen bonding between VD3 and ARG was found to be the main interaction in the binary amorphous system, which can also contribute to solubility enhancement [19,38].

### 3.4. Dissolution Performance of the VD3–ARG Binary Amorphous System

**Figure 7:**
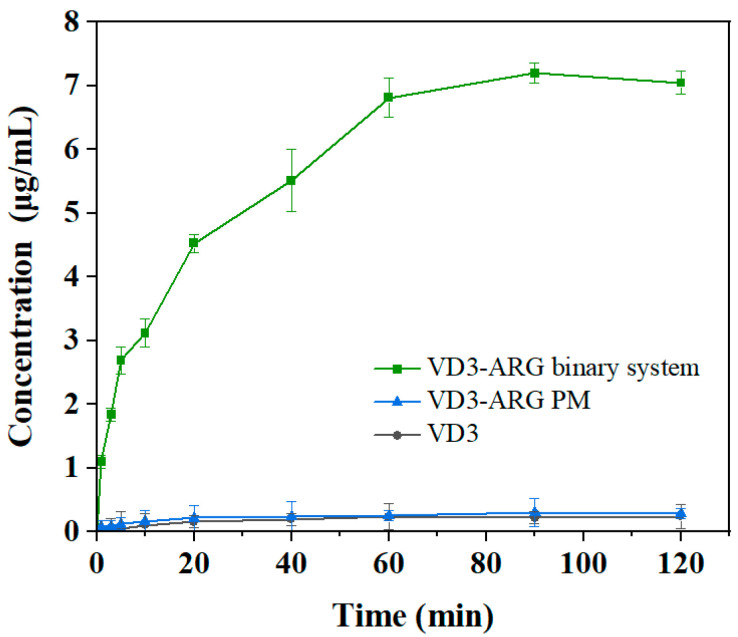
Dissolution curves of VD3, VD3–ARG PM, and the VD3–ARG binary system in ethanol–water (20:80, *v*/*v*) at 37 °C.

The dissolution medium in the dissolution tests was set as ethanol–water (20:80, *v*/*v*) solution to investigate the potential solubility enhancement. The dissolution curves of neat VD3, VD3–ARG PM, and the VD3–ARG binary amorphous system are presented in Figure 7. The solubility of VD3 reached a saturated solubility level at 90 min, with a solubility of 0.223 ± 0.093 μg/mL. The limited solubility improvement in neat VD3 in 20% ethanol–water medium may have arisen from insufficient ethanol concentration to disrupt crystalline nuclei, coupled with excess undissolved VD3 particles inducing recrystallization. In the VD3–ARG PM, the dissolution curve showed no significant changes compared to the neat VD3, with a saturated solubility of 0.305 ± 0.223 μg/mL at 90 min. Compared with pure VD3 and VD3–ARG PM, the superior dissolution behavior was observed in the VD3–ARG binary amorphous system. The saturated solubility of VD3 reached a level of 7.198 ± 0.156 μg/mL in the binary system at 90 min, which was more than 30-fold higher compared to the neat VD3. Although ARG exhibited limited solubility in ethanol–water mixtures, the VD3–ARG binary amorphous system effectively suppressed recrystallization through hydrogen bonding interactions, which remained partially intact despite ethanol’s competitive disruption of molecular associations. In addition, the saturated solubility was maintained for a long time in the binary system, which could significantly increase the area under the dissolution curve. The appearance and maintenance of saturated solubility is attributed to the “spring and parachute effect” in amorphous formulations [39]. Molecular interactions are one of the primary factors to stabilize the amorphous form since the interactions can restrict molecular mobility and reduce the likelihood of crystalline order formation. In the VD3–ARG binary system, hydrogen bonding between VD3 and ARG can inhibit nucleation and crystal growth, thereby preventing the recrystallization of VD3 [20]. Therefore, the formation of a VD3–ARG binary amorphous system could offer a significant improvement in the dissolution behavior of VD3.

## 4. Conclusions

In this study, different types of co-formers were screened to prepare binary amorphous systems with VD3 to improve the dissolution of VD3. Among all the screened VD3–co-former binary systems, the VD3–ARG system showed a significant increase in the solubility of VD3, with an enhancement 80-fold higher compared to neat crystalline VD3. Therefore, ARG was chosen as the ideal co-former to form a binary amorphous system with VD3. The amorphization of the VD3–ARG binary system was confirmed by PXRD diffractograms and DSC thermograms, and the morphology was observed by SEM measurements. Molecular interactions between VD3 and ARG were investigated using FT-IR spectroscopy and MD simulations, and the results revealed three strong hydrogen bonding sites between VD3 and ARG in the binary system. Furthermore, the VD3–ARG binary amorphous system exhibited superior dissolution performance compared to the neat crystalline VD3 and the corresponding physical mixtures. The VD3–ARG binary system achieved a high saturated solubility level (i.e., more than 30-fold higher compared to that of crystalline VD3), and the desired saturated solubility was maintained for a long period (at least 120 min). Therefore, it appears promising to prepare a VD3–ARG binary amorphous system to further develop relevant vitamin D-fortified functional foods or oral supplements.

## Data Availability

The data presented in this study are available on request from the corresponding author. The data are not publicly available to preserve the scientific integrity of the research methodology.

## Supplementary Materials

The following supporting information can be downloaded at: https://www.mdpi.com/article/10.3390/foods14081321/s1, Figure S1: Solubility of VD3-ARG binary system in pure water at pH 3, 7, and 10; Figure S2: PXRD diffractograms of VD3-ARG binary system at room temperature for storage stability evaluation.
