# Supplementary figures and images for: Preparation and Characterization of Vitamin D3-Based Binary Amorphous Systems
Source: Foods. 2025 Apr 11;14(8):1321. doi: 10.3390/foods14081321 (PMC12026574; doi:10.3390/foods14081321)

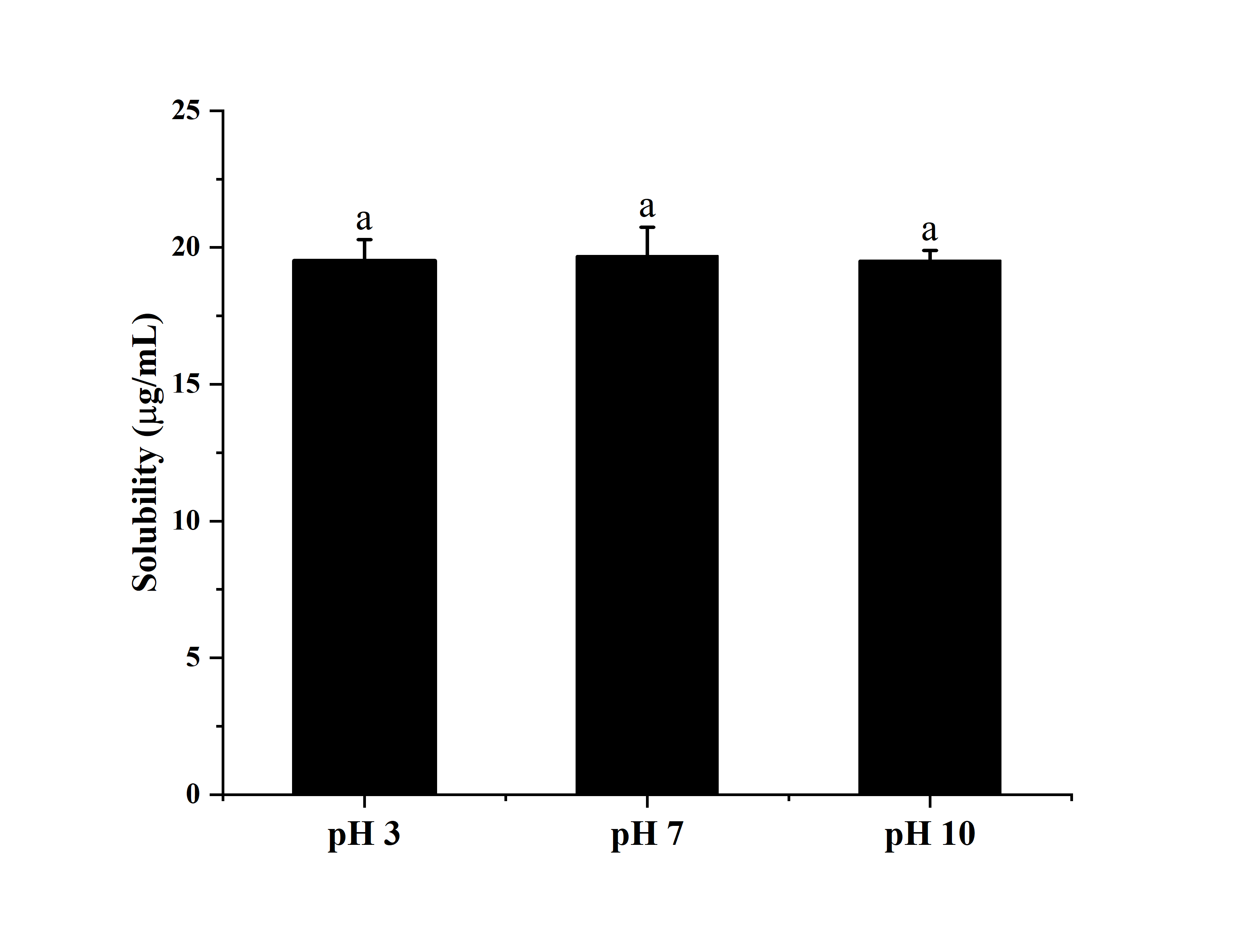

Supplement: Supplementary file 1 [file foods-14-01321-s001.zip › Supplementary Figure S1. Solubility of VD3-ARG binary system in pure water at pH 3, 7, and 10..tif]

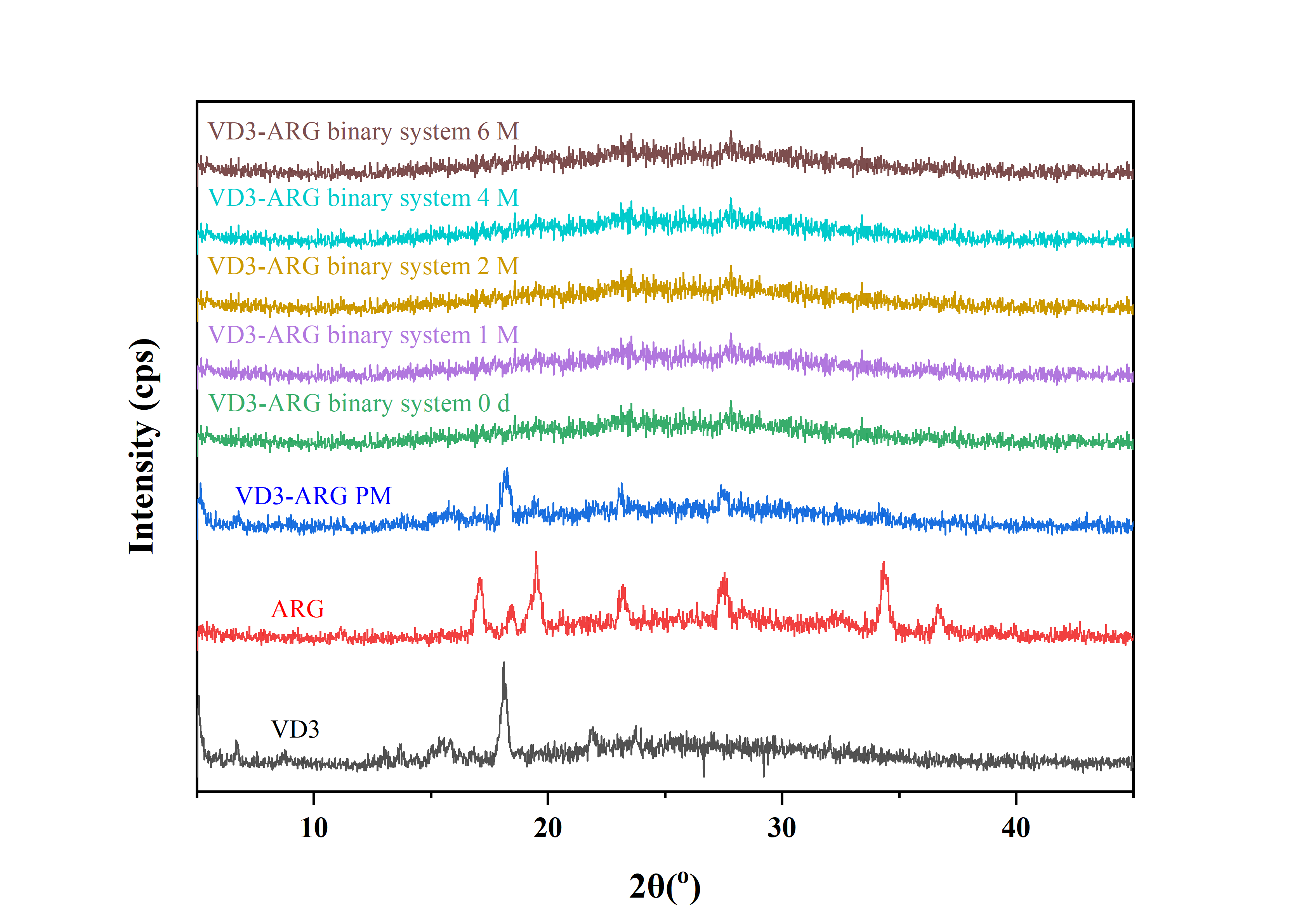

Supplement: Supplementary file 1 [file foods-14-01321-s001.zip › Supplementary Figure S2. PXRD diffractograms of VD3-ARG binary system at room temperature for storage stability evaluation..tif]
